# Medullary nephrocalcinosis in idiopathic hypercalciuria

**DOI:** 10.1002/ccr3.1189

**Published:** 2017-09-25

**Authors:** Abhilash Koratala, Vikrampal Bhatti

**Affiliations:** ^1^ Division of Nephrology, Hypertension and Renal Transplantation University of Florida Gainesville Florida

**Keywords:** hypercalciuria, idiopathic, nephrocalcinosis

## Abstract

Idiopathic hypercalciuria is a metabolic abnormality characterized by excessive calcium excretion in the urine with normal serum calcium levels and is a common risk factor for formation of kidney stones and/or nephrocalcinosis. These patients benefit from a normal‐calcium, reduced‐animal protein, and low‐salt diet, along with a thiazide diuretic.

## Case Description

Nephrocalcinosis is a generalized increase in the calcium content of the kidneys that may occur at a molecular, microscopic or macroscopic level leading to progressive renal damage 1. Nephrocalcinosis commonly involves the renal medulla, and less often, the cortex. In the setting of hypercalciuria, urine concentration and supersaturation lead to calcium crystal deposition in the renal parenchyma. Conditions that are commonly associated with nephrocalcinosis include primary hyperparathyroidism, sarcoidosis, hypervitaminosis D, Milk‐alkali syndrome, distal renal tubular acidosis, medullary sponge kidney, and heavy doses of loop diuretics 2. Herein, we present a classic image of medullary nephrocalcinosis in a patient with idiopathic hypercalciuria.

**Figure 1:**
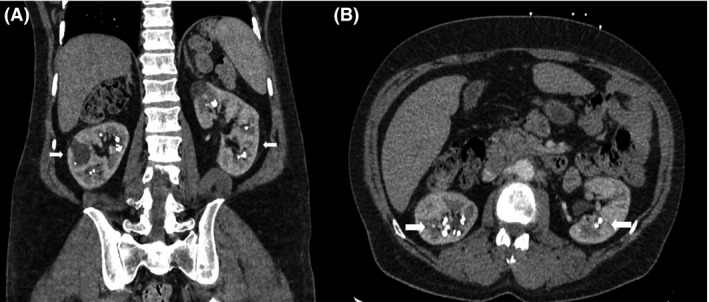
CT scan of the abdomen, transverse, and coronal views, demonstrating bilateral medullary nephrocalcinosis.

**Figure 2:**
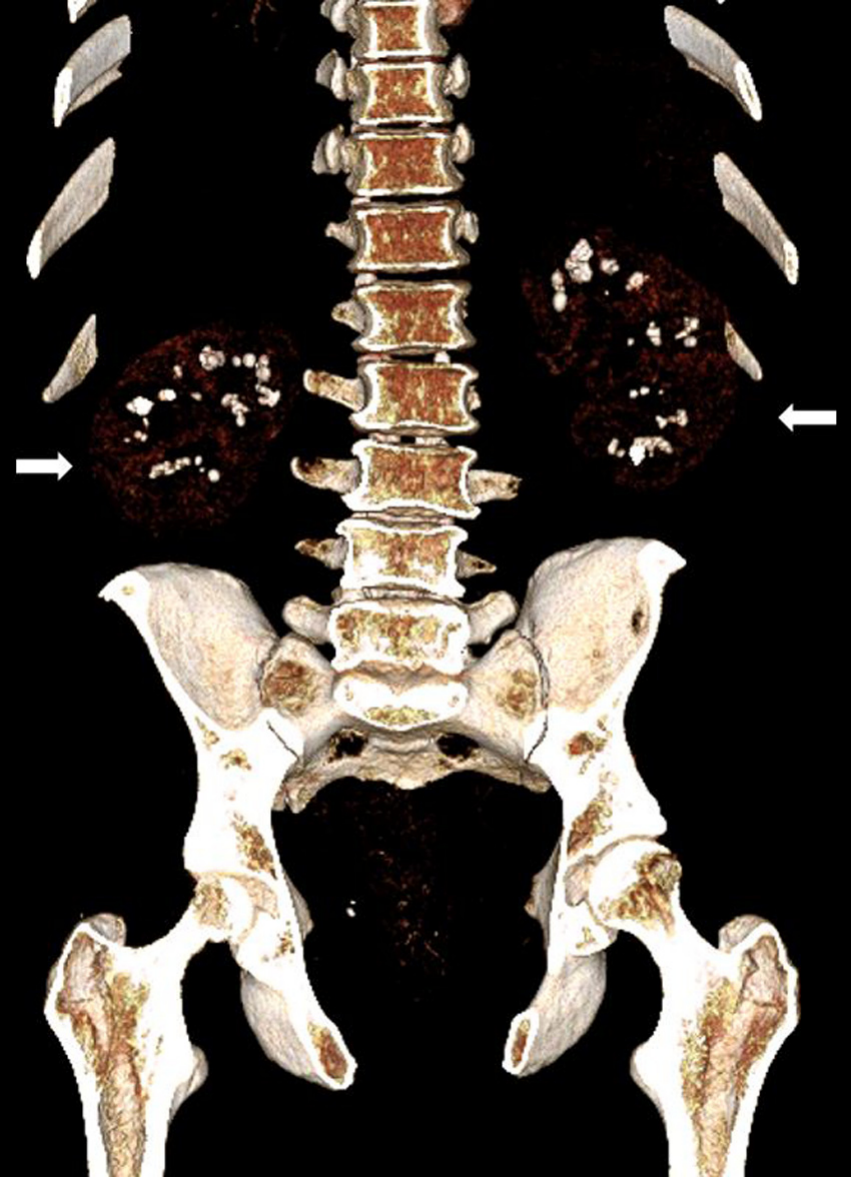
Three‐dimensional reconstruction of the CT scan demonstrating bilateral medullary nephrocalcinosis.

A 34‐year‐old otherwise healthy man was seen for back pain. He apparently had nonspecific back and leg pain for about 2 years and occasional hematuria. CT scan of the abdomen demonstrated calcinosis in the medullary region of both kidneys, suggestive of medullary nephrocalcinosis (Figs. 1 and 2). Laboratory evaluation revealed hypercalciuria with a urine calcium of 557 mg/24 hours. Serum calcium, parathyroid hormone, and Vitamin D levels were within normal limits (9.2 mg/dL, 33 pg/mL, and 22 ng/mL, respectively). There was no evidence for renal tubular acidosis. We diagnosed him with idiopathic hypercalciuria and started on hydrochlorothiazide and low‐sodium diet 3, 4.

## Authorship

All authors made substantial contribution to the preparation of this manuscript and approved the final version for submission. AK: drafted the manuscript. VB: acquired the images, revised the manuscript for critically important intellectual content and approved for final submission.

## Conflict of Interest

None declared.

## Informed Consent

Informed consent has been obtained for the publication of this clinical image.
